# Structural and functional characterization of a frataxin from a thermophilic organism

**DOI:** 10.1111/febs.14750

**Published:** 2019-01-30

**Authors:** Masooma Rasheed, Mostafa Jamshidiha, Rita Puglisi, Robert Yan, Ernesto Cota, Annalisa Pastore

**Affiliations:** ^1^ King's College London UK; ^2^ UK Dementia Research Institute at King's College London UK; ^3^ Department of Life Sciences Imperial College London South Kensington UK; ^4^ University of Pavia Italy

**Keywords:** Friedreich's ataxia, iron–sulfur clusters, structural biology

## Abstract

Frataxins form an interesting family of iron‐binding proteins with an almost unique fold and are highly conserved from bacteria to primates. They have a pivotal role in iron–sulfur cluster biogenesis as regulators of the rates of cluster formation, as it is testified by the fact that frataxin absence is incompatible with life and reduced levels of the protein lead to the recessive neurodegenerative disease Friedreich's ataxia. Despite its importance, the structure of frataxin has been solved only from relatively few species. Here, we discuss the X‐ray structure of frataxin from the thermophilic fungus *Chaetomium thermophilum*, and the characterization of its interactions and dynamics in solution. We show that this eukaryotic frataxin has an unusual variation in the classical frataxin fold: the last helix is shorter than in other frataxins which results in a less symmetrical and compact structure. The stability of this protein is comparable to that of human frataxin, currently the most stable among the frataxin orthologues. We also characterized the iron‐binding mode of Ct frataxin and demonstrated that it binds it through a semiconserved negatively charged ridge on the first helix and beta‐strand. Moreover, this frataxin is also able to bind the bacterial ortholog of the desulfurase, which is central in iron–sulfur cluster synthesis, and act as its inhibitor.

**Abbreviations**
Ct: *Chaetomium thermophilum*
FRDA: Friedreich's ataxia
isc: iron–sulfur complex
PDB: protein data bank
*T*_m_: melting temperature

## Introduction

Iron–sulfur clusters are inorganic cofactors thought to be present already in the protocell and to have provided the most ancient response to the problem of storing the toxic and yet essential iron and sulfur elements in living cells 1. In proteins, they are usually coordinated by cysteine and/or histidine residues. Thanks to their favorable redox potential, iron–sulfur cluster proteins cover also in the modern cell essential functions in catalysis, electron transfer, and regulation of gene expression 2, 3. Iron–sulfur clusters also act as sulfur donors in biotin and lipoic acid cofactor biosynthesis. Their synthesis and assembly into the target proteins is a complex and tightly regulated process under the control of evolutionary conserved machines, which were all discovered around year 2000 4, 5.

In modern bacteria, the machines able to perform cluster assembly are from the nif (nitrogen fixation, ^Nif^iscA‐nifSU), isc (iron–sulfur complex, iscRSUA‐hscBA‐fdx) and suf (mobilization of sulfur, sufABCDSE) operons. Amongst these, the most universal one is the isc operon (reviewed in 6, 7) whose gene products form an intricate network of interactions. The two main components are the desulfurase IscS which converts cysteine into alanine and the scaffold protein IscU which acts as a transient acceptor of the cluster. This is then transferred to other acceptors which will distribute it around. Most of the isc components have direct mitochondrial orthologues in eukaryotes allowing direct comparison between the prokaryotic and eukaryotic systems. Depletion or mutation of several of the eukaryotic components is associated with human diseases which manifest with impairments of iron homeostasis including increased cellular iron uptake and mitochondrial iron overload (reviewed in 8). Examples include pathologies associated with neurodegeneration, myopathies and hematology. Among these, an important neurodegenerative disease caused by partial silencing of the mitochondrial protein frataxin is Friedreich's ataxia (FRDA), an autosomal recessive neurodegenerative disease with occurrence in 1:50 000 and onset usually before 25 years of age 9. FRDA is associated with iron accumulation and oxidative stress which produce organ and metabolic damage. Death is usually caused by cardiac failure or diabetes‐related complications. Frataxin is a highly conserved iron‐binding protein that is present both in prokaryotes and eukaryotes. It regulates the rates of cluster formation and possibly acts as an iron donor through an interaction with the two central components of iron–sulfur cluster formation, the desulfurase and the scaffold protein 10. The structures of frataxins from different species ranging from bacteria, to yeast, plants and humans have been published. They have very similar features with a globular domain preceded, only in eukaryotes, by a low conserved tail which contains the mitochondrial import signal. The surface of interaction with the desulfurase involves a semiconserved negatively charged ridge 11.

Here, we report the crystal structure of frataxin from the fungus *Chaetomium thermophilum* (Ct). This organism is a thermophile and is considered a good source of proteins that are more stable than their mesophile counterparts 12, 13, making crystallization and other structural studies easier. We find that, while sharing most of the properties of the other frataxin structures, Ct‐frataxin has interesting peculiarities which make the system an excellent candidate for future studies of the folding determinants of the frataxin family.

## Results

### Ct‐frataxin has a stability comparable to that of human frataxin

**Figure 1:**
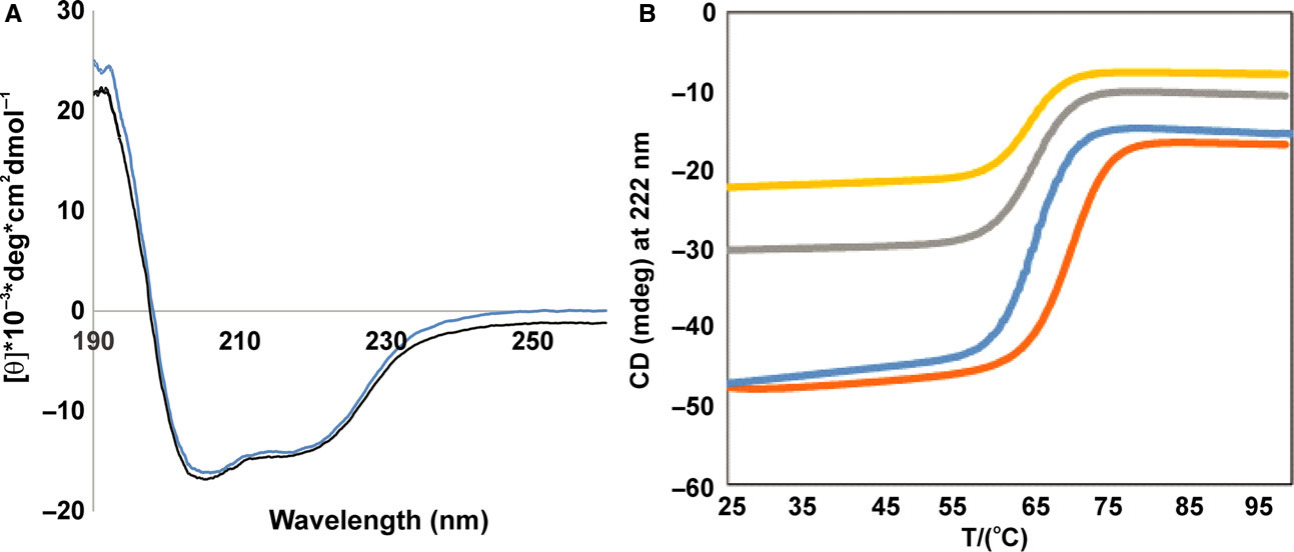
Characterization on the fold and stability of Ct‐frataxin. (A) Far‐UV CD spectra at 25 °C showing all the features of a well folded protein. The two spectra were collected before (blue) and after (black) heating the sample at 90 °C. The spectra were recorded with a 22 μm concentration sample in 20 mm sodium phosphate at pH 7.4. (B) Thermal denaturation curves of 22 μm of Ct‐frataxin in 10 mm Hepes buffer at pH 7.4 in the presence of 10 mm CaCl_2_ (orange), 1 mm CaCl_2_ (blue), 150 mm KCl (grey) and 100 mm NaF (yellow).

Not knowing the exact domain boundaries of the mature form, we expressed the protein using boundaries approximately corresponding to those of the conserved C‐terminal domain in human frataxin (residues: 87–210 in Ct‐frataxin). After protein purification, the size exclusion chromatography profile of Ct‐frataxin indicated a highly pure monomeric protein (data not shown). We checked Ct‐frataxin for its fold and stability by CD. The spectrum has a α‐helical‐dominated appearance with two minima at 208 and 222 nm (Fig. 1A). The thermal unfolding is completely reversible as demonstrated by rerecording a spectrum at room temperature after having heated the sample to 95 °C. This indicates solubility and lack of aggregation at high temperature. The protein undergoes a highly cooperative T‐jump which is well fitted assuming a two‐state transition. The midpoint of the transition is around 58 °C in the absence of added salt (Fig. 1B). This value is intermediate between the stability of the bacterial and the human orthologues 14. Mono and divalent cations stabilise the protein by up to 10 °C making this protein more stable than human frataxin which has a melting point around 58–65 °C, depending on the absence or presence of cations (Table 1). This behavior is similar to what is described in Adinolfi *et al*., 14, where the effect of different cations on the stability of bacterial frataxin CyaY was investigated. This evidence was interpreted as a hint to confirm that frataxins are cation‐binding proteins. What we now know is that frataxins bind primarily to iron, but also several other cations even though with lower affinity 15, 16. Since the affinity to iron is in the micromolar range and we do not know the exact stoichiometry because the binding is purely electrostatic, we used an excess of calcium. We thus interpret the lack of variations of the melting temperature (*T*_m_) between 1 and 10 mm calcium concentrations as an indirect evidence that the binding site is completely saturated already at 1 mm.

**Table 1:** Buffer conditions used for CD measurements of Ct‐frataxin protein

Buffers	*T*_m_ (°C)
10 mm CaCl_2_, 10 mm Hepes, pH 7.4	69.8
1 mm CaCl_2,_ 10 mm Hepes, pH 7.4.	65.7
100 mm NaF, 10 mm Hepes, pH 7.4	65.5
150 mm KCl, 10 mm Hepes, pH 7.4	66.3
20 mm NaH_2_PO_4_ pH 7.4	58.1

### Solving the X‐ray structure of Ct‐frataxin

We next solved the X‐ray structure of Ct‐frataxin by molecular replacement using the coordinates of human frataxin at 1.3 Å [protein data bank (PDB) code: 3s4m] (Table 2). Ct‐frataxin crystals were produced only when the protein stock solution was dialyzed against a buffer with pH close to the protein isoelectric point (4.9). Although the size of the crystals was 20*40*30 μm, initial diffraction on the I04‐1 beam line at Diamond Light Source (Oxford, UK) resulted only in datasets at 3 Å resolution. Eventually a 2.1 Å dataset was acquired after diffracting a crystal at the I24 micro focus beam line. The anisotropic Delta‐B, that is, the difference between the two most extreme principal components of the anisotropic B factor along different directions in reciprocal space, was 12.2 Å^2^ indicating that the data are not anisotropic. Intensity statistical analysis indicated that the crystal is not twinned. Although human frataxin was used as the model for molecular replacement, a solution was obtained only when the loops between Ala104 and Asp124, Arg165 and Leu182, and Lys195 to the C‐terminus of human frataxin were removed from the search model.

**Table 2:** Data collection and refinement statistics

Total oscillation	100°
Oscillation per image	0.2°
Wavelength	0.96861
Resolution range	52.11–2.034 (2.106–2.034)^a^
Space group	P 41 21 2
Cell dimensions	
*a*, *b*, *c* (Å)	89.058, 89.058, 185.62
α, β, γ (°)	90, 90, 90
Total reflections	354 746 (17 646)
Unique reflections	48 810 (4784)
Multiplicity	7.3 (6.5)
Completeness (%)	100 (100)
Mean *I*/sigma(*I*)	15.1 (2.1)
Wilson *B*‐factor	33.45
*R*‐merge	0.079 (0.940)
*R*‐means	0.086 (1.011)
CC1/2	1.0 (0.8)
Reflections used in refinement	48 738 (4781)
Reflections used for *R*‐free	2440 (234)
*R*‐work	0.2188
*R*‐free	0.2596
Number of nonhydrogen atoms	5387
Macromolecules	5209
Ligands	28
Protein residues	684
RMS (bonds)	0.0147
RMS (angles)	1.6768
Ramachandran	
Favored (%)	99
Allowed (%)	0.6
Outliers (%)	0
Rotamer outliers (%)	0.56
Average *B*‐factor	41.59
Macromolecules	41.69
Ligands	42.11
Solvent	38.08

a Values in parentheses are for highest‐resolution shell.

**Figure 2:**
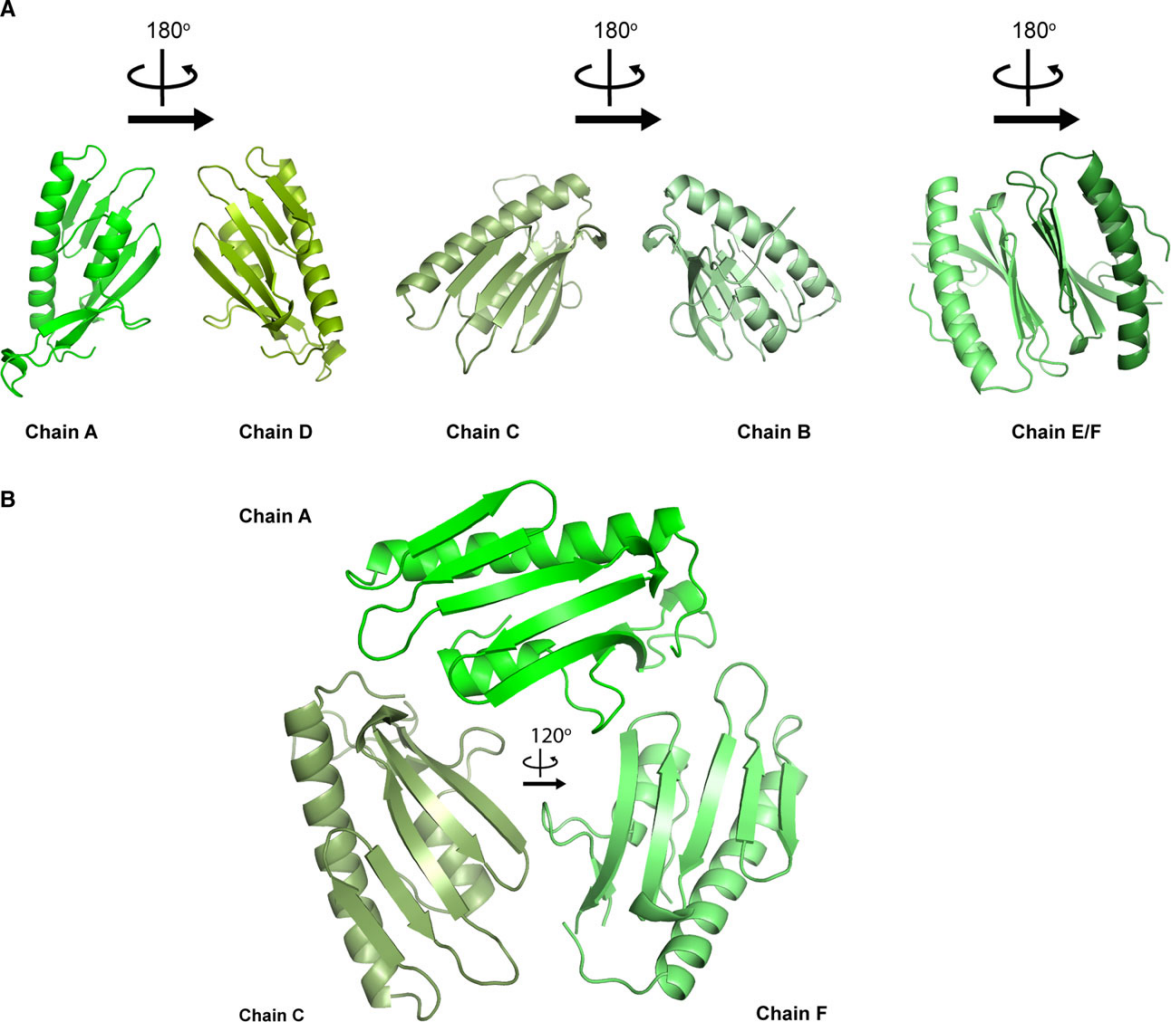
Symmetry of the hexamer in the crystallographic unit. (A) The three possible crystallographic dimers. (B) Ternary symmetry between protomers. The different protomers are shown in different shades of green.

Understanding the internal symmetry is not trivial. The structure contains six protein molecules in each asymmetric unit, with interactions between the tips of chain A with C and E, B with D and E, F with C and D according to noncrystallography symmetry twofold axes. More extensive interactions are between chains AD, BC, and especially EF which pack against each other in pairs through the β‐sheet surfaces (Fig. 2A). A threefold axis can be observed between CDE and ABF (Fig. 2B). Comparing the model built for chains A–F reveals that the electron density is recognizable for the loop between Ile166‐Glu181, whereas no electron density was entirely visible for chain D and not present at all in chains E and F. Models could only be built up to Leu203 for chains A, B and D and up to Val205 for chains C, E and F. Superposition of the six protomers results in 1 Å average root mean square deviation (r.m.s.d.). The structure of each protomer comprises the usual frataxin fold which consists of a sheet with seven strands flanked by two helices, α1 and α2 11.

### The structure of Ct‐frataxin reveals features distinct from other orthologues

**Figure 3:**
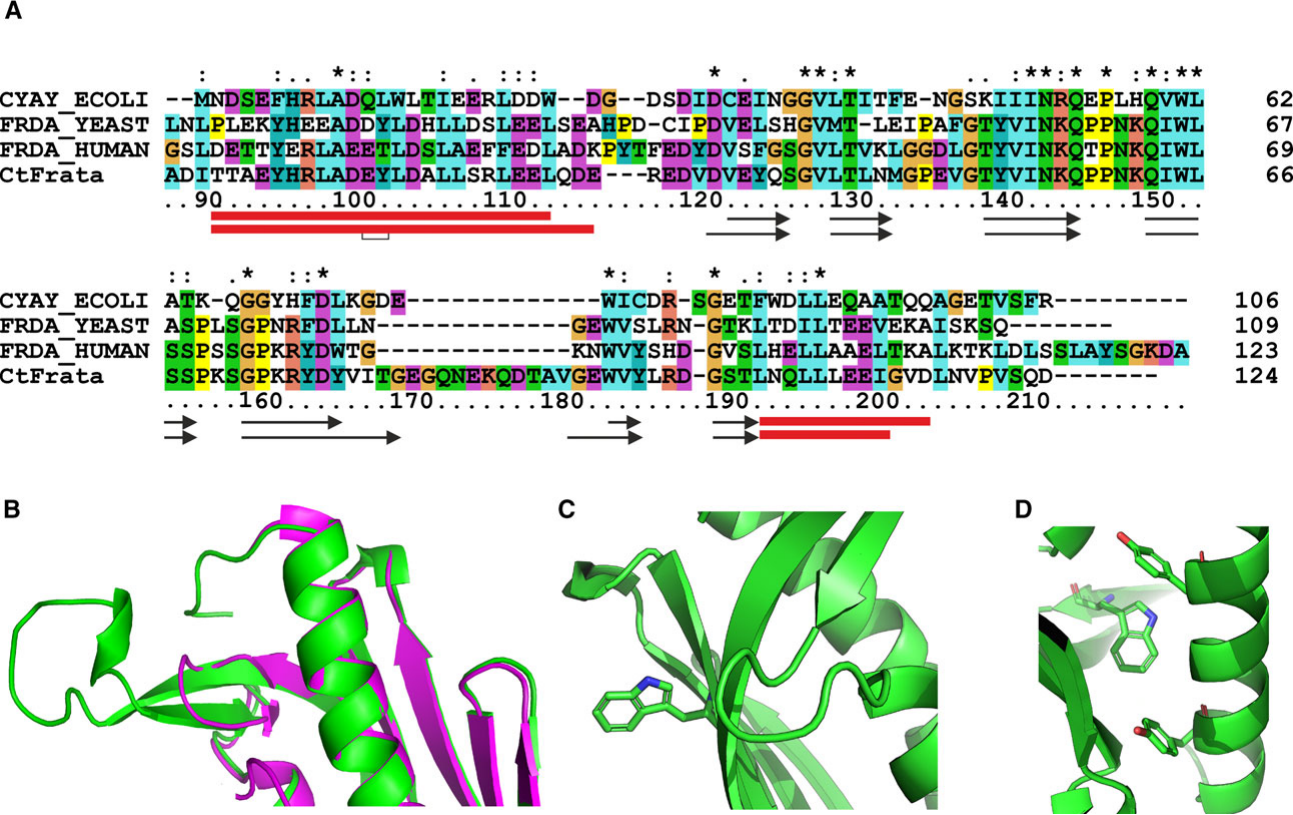
Structural features of Ct‐frataxin as compared to other orthologues. (A) Multiple alignment of frataxin sequences from different organisms. The alignment was achieved by clustalx. Asterisks, semicolons and dots indicate complete and partial conservation. The secondary structures of human and Ct‐frataxins are indicated on the bottom with red boxes and black arrows for helices and strands, respectively. (B) Details showing the superposition of the loop β5/β6 in human (magenta) and Ct‐frataxins (green). (C) Details of the surrounding of the exposed Trp151. (D) Close‐up of the environment of the buried Trp181.

*Chaetomium thermophilum*‐frataxin reveals several peculiar features as compared to the sequences and structures of other members of the frataxin family. From a multiple alignment based on structural superposition it is clear that the length of two loops is different in Ct‐frataxin as compared to other sequences: the loop between α1 and β1 that has a three amino acid deletion as compared to human frataxin and the loop between β5 and β6 that contains a 13 amino acid insertion in Ct‐frataxin (Fig. 3A). The position of the secondary structure elements is in an excellent agreement with those of other orthologues except for the length of α1 and α2 that are, respectively, one turn longer and one turn shorter in Ct‐frataxin as compared to human frataxin. β5 and β6 are also longer in Ct‐frataxin.

Superposition of human (PDB code 3s4m) and Ct‐frataxin on the backbone residues 95–115, 123–163, 172–190 and 94–114, 119–159, 181–199, respectively, results in a r.m.s.d. of 0.74 Å with the side chains of the two helices well superposed (Fig. 3B). As a result of the insertion between α1/β1, the conformation of the corresponding tight turn is slightly different in the two structures. The β5 and β6 insertion, which contains small or charged amino acids, protrudes out into solution without forming any defined secondary structure with the exception of a short turn where the chain bends to invert its direction (Fig. 3B).There are only two tryptophans in Ct‐frataxin, both very conserved in the family, Trp151 and Trp182. Trp151 in β4 corresponds to Trp155 in human frataxin. In all frataxin structures, this residue is exposed to the solvent and participates to interaction with the desulfurase‐scaffold complex 11, 17 (Fig. 3C). The second tryptophan at the beginning of β6 has a structural role being buried and forming a stacking interaction with a conserved tyrosine (Fig. 3D). We cannot say much about the C‐terminus of the protein because of lack of definition in the electron density. The beginning of α2 is well defined with two contiguous leucines well aligned with those of human frataxin. Lack of definition of the C‐terminus in Ct‐frataxin could be the consequence of crystal heterogeneity and would explain why the stability of this protein is high but overall comparable to the human orthologue despite the fact that the protein comes from a thermophile organism.

**Figure 4:**
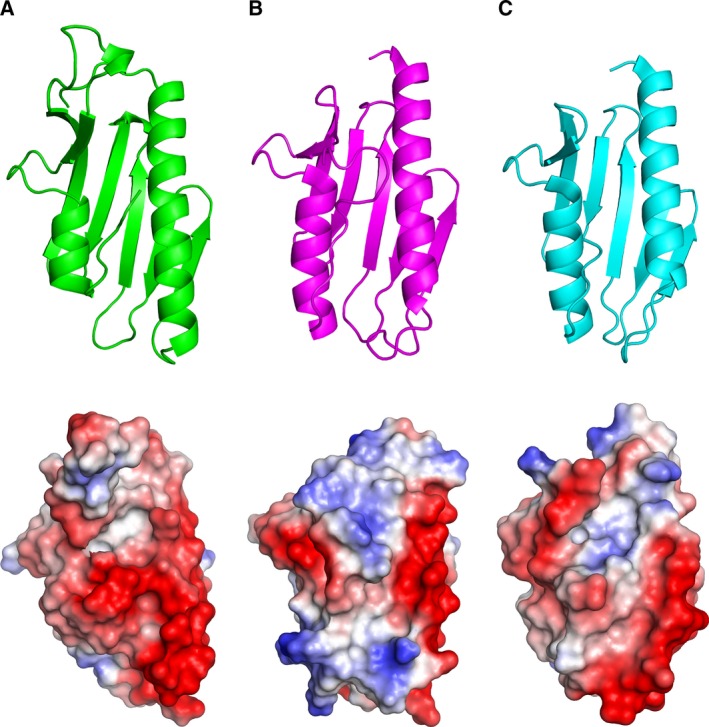
Structural features of Ct‐frataxin. Top: Comparison between the structures of Ct‐frataxin. (A), human (3s4m) (B) and bacterial (1ew4) (C) frataxins. Bottom: electrostatic surfaces of frataxin in the same order as in the top.

The structure of Ct‐frataxin looks overall less compact and symmetric than the other orthologues (Fig. 4, upper panels). The surface charge is mostly negative as also expected from the isoelectric point of the protein and as observed also in other orthologs (Fig. 4, lower panels). The most negatively charged region contains the semiconserved negatively charged ridge on α1 11.

### Solution NMR supports a shorter α2 and provide information on protein dynamics

**Figure 5:**
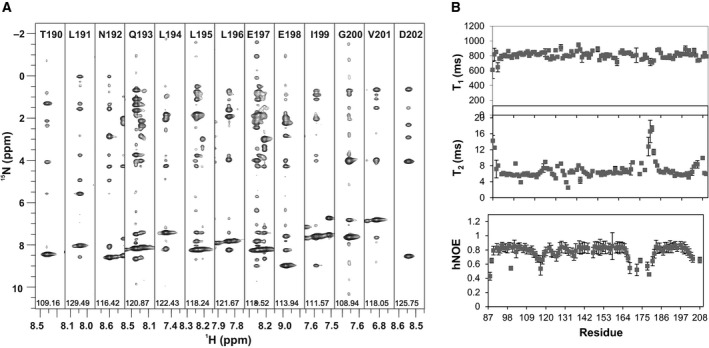
Dynamics of Ct‐frataxin in solution. (A) Strips from a ^15^N NOESY‐HSQC for the residues 191–202. Sequential HN–HN connectivities define the limits of α2 from Leu192 to Val202. (B) *T*_1_ and *T*_2_ relaxation times. The spectra were collected at 37 °C and 800 MHz.

To clarify the conformation of the C‐terminus of Ct‐frataxin and compare the features observed in the crystal with those of the protein in solution, we resorted to NMR. We had previously assigned the NMR spectrum of Ct‐frataxin 18. We thus recorded a 3D ^15^N NOESY‐HSQC spectrum to check the position and extension of α2. The presence of matching HN–HN connectivities in sequential strips of the spectrum clearly indicates that the helix extends from residues Leu192 to Asp204 according to what is expected from sequence alignment (Fig. 5A). The helix breaks beyond this residue. These results are further backed up by the secondary chemical shift analysis previously published 18.

We used NMR also to obtain information on the dynamics of Ct‐frataxin. The profile of *T*_1_, *T*_2_ and hetero‐nuclear overhauser enhancements (NOEs) is rather flat as expected for a well ordered and stably folded protein with average *T*_1_ and *T*_2_ values at 800 MHz and 37 °C and 7 ms, respectively. There are, however, some important deviations (Fig. 5B). Large deviations of the *T*_1_ values from the average values were observed around the insertion between β5 and β6. This evidence indicates that the loop is intrinsically flexible as also supported by the temperature factors in the crystal structure which are appreciably higher and the truncation in this region observed in chain D. Also the N‐ and C‐termini have values which differ from the average although overall the protein is stably structured. Smaller deviations are observed for residues 110–140 which host α1 and β1. The correlation time calculated from the *T*_1_/*T*_2_ ratios is 6.5 ns at 37 °C. This value is well in agreement with that expected for a protein of 13.5 kDa supporting the hypothesis that Ct‐frataxin, like all the other orthologues, is a monomer in solution in the absence of iron excesses 19. This suggests that the assembly observed in the crystal does not exist in solution and is likely to be a crystallographic artefact.

### Ct‐frataxin binds iron in the same binding site as other orthologues

**Figure 6:**
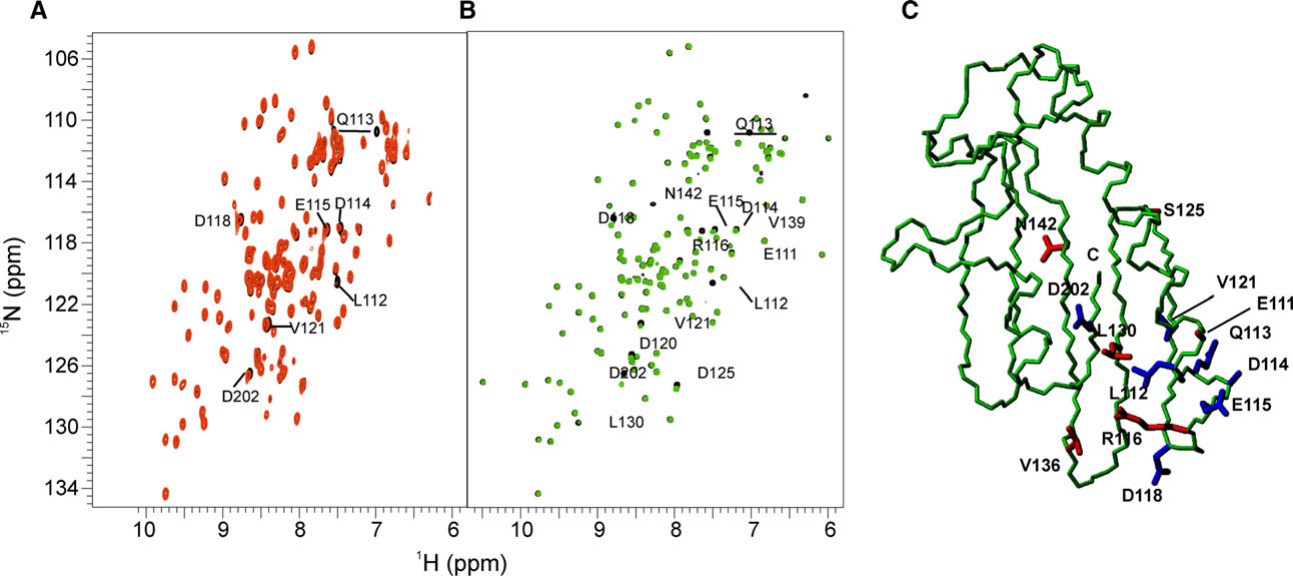
Titration of Ct‐frataxin with iron. (A) Titration with Fe^2+^ at 1 : 1 molar ratio. (B) The same with Fe^3+^. The reference spectra are in black, the spectra after adding Fe^2+^ and Fe^3+^ are in red and green, respectively. The experiments were carried out at 600 and 950 MHz, respectively and 37 °C. (C) Map of the residues affected. In blue are the side chains of residues affected by both Fe^3+^ and Fe^3+^. In red are the side chains of residues only affected by Fe^3+^.

We then used NMR to test if Ct‐frataxin binds to iron and to map the potential binding sites. ^1^H,^15^N labeled Ct‐frataxin was titrated with increasing quantities of (NH_4_)_2_Fe(SO_4_)_2_. As in previous cases 10, 17, the experiment was carried out aerobically to circumvent the necessity of having a glove box close enough to the NMR spectrometer. Almost all the effects observed occurred in the α1/β1 loop and in the surrounding region (Fig. 6A). The amide of Leu112 in α1 disappeared. Asp114, Glu115, Asp118 and V121 in the β1 sheet shifted. Other peaks (Leu109, Asp111 and Glu122) followed a pattern of intermediate/fast exchange with moderate chemical shift variations in both ^1^H and ^15^N dimensions when exposed to lower or equivalent molar ratios of iron to protein. When Fe(III) was used (FeCl_3_) we observed effects on the same resonances and, in addition, on a few other nearby residues (Glu111, Arg116, Asp120, Asp125, Leu130, and Val136; Fig. 6B). Asn142 on β3 disappeared but the peak was very weak and broad to start with. Asp202 is in the C‐terminal tail which sits very close and might have been affected because of its proximity to the binding site. With both cations, the residues affected clustered on the same surface of the protein which corresponds to the iron‐binding region of bacterial and human frataxin 15, 16, 20 (Fig. 6C). This region comprises the semiconserved negatively charged ridge originally identified in human frataxin 11. We can thus conclude that Ct‐frataxin has a weak capacity to bind to iron as observed for other orthologues.

### Ct‐frataxin binds to IscS and behaves as an inhibitor of bacterial IscS

**Figure 7:**
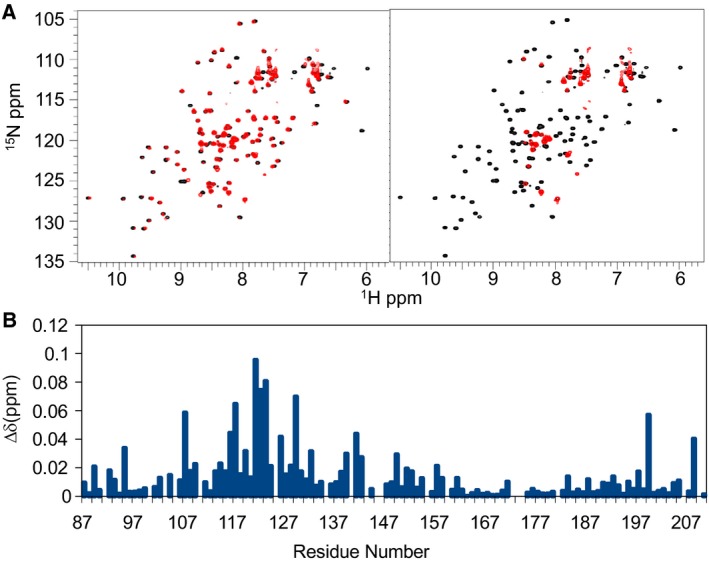
Ct‐frataxin is able to bind and inhibit the enzymatic function of bacterial IscS. (A) Titration of labeled Ct‐frataxin with unlabeled bacterial IscS at a 1 : 0.5 Ct‐frataxin : IscS molar ratio (left) and at a 1 : 3 Ct‐frataxin : IscS molar ratio (right). (B) Chemical shift perturbation observed at low Ct‐frataxin : IscS ratios plotted along the frataxin sequence.

To test whether Ct‐frataxin behaves as other frataxins, we wanted to check if it is competent in regulating the enzymatic formation of iron–sulfur clusters. However, we could not produce an active desulfurase from Ct we mixed Ct‐frataxin with the bacterial desulfurase and both bacterial and Ct‐IscU. We first checked by NMR if Ct‐frataxin was able to bind the *Escherichia coli* scaffold protein IscU and the desulfurase IscS. A significant loss of resonance intensity and broadening due to intermediate/fast exchange was observed in different regions of the HSQC of ^15^N labeled Ct‐frataxin upon titration with increasing quantities of unlabeled IscS. Already at a 1 : 0.5 Ct‐frataxin : IscS molar ratio a number of resonances disappear or broaden significantly (Fig. 7A). Other resonances shift. The spectrum disappears almost completely at a 1 : 3 Ct‐frataxin : IscS molar ratio (Fig. 7B). The remaining resonances mostly belong to N‐terminal residues suggesting that this region is more flexible or exposed. When we reported the chemical shift perturbations observed at low IscS : Ct‐frataxin molar ratios as a function of the sequence, we observed the largest effects clustered around α1 and β1 (Fig. 7C). This region is the same observed to be affected by the interaction between the bacterial frataxin and IscS proteins 17. These results indicate that, despite dealing with a heterologous system, Ct‐frataxin is capable of binding with appreciable affinity bacterial IscS with a surface of interaction similar to that observed for the bacterial orthologue. Conversely, when we titrated ^15^N labeled Ct‐frataxin with unlabeled IscU up to a 5 molar excess of the latter, we did not observe any variation in the spectrum (data not shown). This result is not surprising because no direct interaction has yet been reported between frataxin and the scaffold protein in the absence of iron.

**Figure 8:**
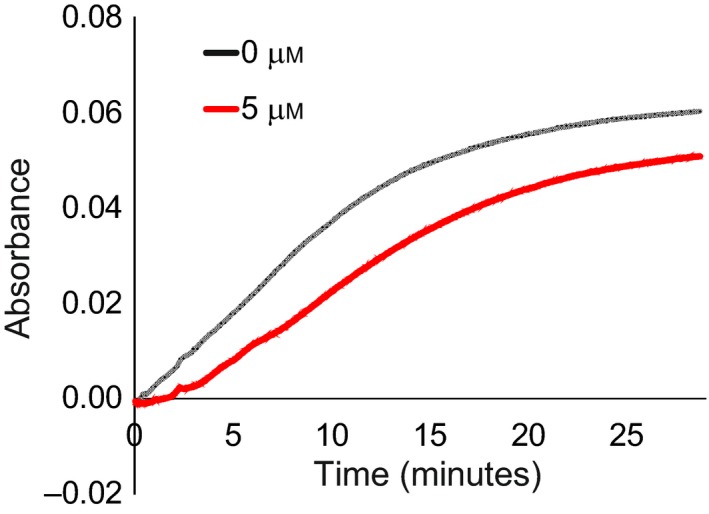
Kinetics of the enzymatic formation of iron–sulfur clusters on the scaffold protein IscU mediated by the desulfurase IscS followed by UV absorbance. The two curves were obtained in the absence of Ct‐frataxin (black) and in the presence of 5 μm Ct‐frataxin.

Finally, we tested the properties of Ct‐frataxin in iron–sulfur cluster formation. We followed, under strict anaerobic and reducing conditions, the kinetics of IscS‐mediated enzymatic formation of iron–sulfur clusters on IscU measuring the absorbance as a function of time for increasing concentrations of Ct‐frataxin (0–10 fold). We used concentrations of all reagents similar to those chosen in previous studies 10, 17, 21. We observed an inhibitory effect as compared to the kinetics in the absence of frataxin (Fig. 8).

## Discussion

The interest for frataxin, a protein previously totally ignored, was originally raised in 1996 when the protein was linked to FRDA, a rare but lethal neurodegenerative disease 22. Since then, frataxin was studied and linked to the essential and conserved machine that assembles iron–sulfur clusters, prosthetic groups of crucial importance for the cell 23. In more recent years, it has become clear that frataxin plays an important role as an interactor and regulator of the desulfurase that provides the sulfur necessary to build iron–sulfur clusters 10, 17, 24, 25. Despite this, the number of structures from different species are currently very limited comprising only five different species: *E. coli, Helicobacter pylori*, the psychrophile *Psychromonas ingrahamii, Saccharomyces cerevisiae* and *Homo sapiens*. We have added here the structure of a thermophile, the Ct fungus, to this list. This eukaryotic organism lives on dungs or compost and has a high temperature tolerance (60 °C) with an optimal growth temperature of 50–55 °C. Ct is increasingly used in structural biology because, thanks to their thermal resistance, the proteins from this organism are excellent eukaryotic models for crystallization. Indeed, Ct‐frataxin proved overall a good protein to obtain crystals even though some of them did not diffract at the necessary resolution. Structural comparison of Ct‐frataxin with other orthologues reveals interesting features in this protein which make it quite distinct. Apart from the β5/β6 insertion, the main difference in Ct‐frataxin is in the last helical turn of α2 which unfolds and forms a loop which inserts between the two helices still preserving the overall fold. Unfolding is presumably due to the presence of Gly201 which replaces longer chains able to form interactions with the C‐terminus and stabilise the helix. What we observed in the crystal could of course only represent the most stable conformation under the crystallization conditions. When, however, we extended the investigation in solution we observed features that strongly suggest that the length of α2 is not just an artefact of crystallization: solution studies conclusively demonstrate that the conformation observed in the crystal is present also in solution. These results indicate that what we observe in the crystal is an intrinsic feature of frataxin from this species.

Such a feature makes the length of the region following α2 up to the C‐terminus longer than the corresponding region of human frataxin. This is at variance with the sequence alignment of the frataxin family which would suggest a slightly longer than in the *E. coli* orthologue but appreciably shorter than in human frataxin (Fig. 3). This region of the protein is important because we previously demonstrated that the stability of *E. coli*, yeast and human orthologues strongly depends on the length of the C‐terminal insertion between the two helices 14. The behavior of the C‐terminus could thus explain why the protein is stable but not significantly more stable than the human orthologue. Shortening of α2 could have only minor relevance to function since all of the interactions currently known map on α1 and on the sheet. It would be interesting to identify partners specific for Ct interacting with the β5/β6 insertion.

Compared to other frataxins, Ct‐frataxin also seems to be capable to bind iron with low affinity in the corresponding surface observed in other orthologues, supporting the strong evolutionary conservation of this function. More surprising is that Ct‐frataxin binds to the desulfurase IscS from *E. coli*. This is not entirely new: thanks to the high level of sequence conservation, it is possible to mix proteins from different organisms and observe interactions even though with lower affinity than for proteins from the same organism 26, 27. Mixing experiments of *E. coli* and *H. sapiens* proteins had previously demonstrated that frataxins and the scaffold protein are interchangeable among species whereas desulfurases are not 27. Furthermore, the prokaryotic desulfurases are fully active, the eukaryotic ones are inactive and require to be stabilized by the additional Isd11 protein and activated by frataxin 28, 29. This was explained by a different assembly of the IscS‐IscU complex as compared to the eukaryotic Nfs1‐Isu complex 25, 30. We observed a consistent behavior: Ct‐frataxin behaves as an inhibitor of the enzymatic cluster formation catalyzed by IscS. This confirms the hypothesis that the difference between the prokaryotic and eukaryotic iron–sulfur assembly system solely depends on the nature of the desulfurase or, more likely, on the presence in the system of the eukaryotic‐specific Isd11 protein.

Taken together, our results add new information on the frataxin family which may eventually be used to gain a fuller understanding of these fascinating and still so complex proteins.

## Materials and methods

### Protein production

The conserved domain from Ct‐frataxin (Ala87‐Asp210) was expressed in *E. coli* BL21 (DE3) cells at 37 °C in 2YxT media following the overnight induction with isopropyl‐β‐d‐thiogalactopyranoside at 18 °C and purified as previously described 18. For NMR purposes, ^15^N‐uniformly labeled protein samples were prepared, using M9 culture media and supplemented with MgSO_4_, CaCl_2_, biotin, thiamin, freshly prepared FeSO_4_ and ^15^N‐ammonium sulfate as the sole nitrogen source. The cell pellet was collected by centrifuging the sample at 3220 ***g*** for 10 min, sonicated and centrifuged again at 21 000 ***g*** for 45 min. The His‐affinity tag was removed by overnight incubation with TEV protease. The protein was further purified by a second reverse Ni‐affinity step followed by FPLC size‐exclusion chromatography (Superdex 75; GE Healthcare, Chicago, IL, USA) as previously described 11. Fractions were collected and concentrated in aliquots suitable for the various experiments. The concentration of the Ct‐frataxin domain samples was measured by UV absorbance at 280 nm using a calculated extension coefficient of 21 430 m ^−1^·cm^−1^ and a theoretical molecular weight of 14 130 g·mol^−1^. Bacterial IscU and IscS were produced as previously described 10.

### CD

Far‐UV CD spectra (190–260 nm) were recorded on Jasco J‐815 CD equipped with a Jasco CDF‐4265/15 Peltier unit with a cell holder thermostated by circulating water from a Neslab RTE‐111 water bath. Spectral measurements were carried out using Ct‐frataxin protein concentration 22 μm in different buffer conditions using QS quartz 1 mm demountable cuvettes (Hellma analytics, Müllheim, Germany). Baseline correction was obtained by subtraction of the appropriate buffer spectrum. Thermal unfolding curves were obtained by monitoring the ellipticity at 222 nm with a heating rate of 2 °C·min^−1^ in the temperature range 25–95 °C. The apparent *T*_m_ was calculated assuming a two‐state mechanism of unfolding and using nonlinear regression analysis as described by in 31.

### Protein crystallization

*Chaetomium thermophilum*‐frataxin protein was dialyzed against 100 mm NaCl, 10 mm sodium acetate at pH 6.5 and subsequently concentrated up to 10 mg·mL^−1^. Sitting‐drop vapor diffusion crystallization experiments with Ct‐frataxin were set up with a Mosquito Robot (TTP Labtech, Melbourn Science Park, Melbourn, UK) at 20 °C. Search for crystallization conditions was performed using ~ 1000 commercial conditions from Molecular Dimensions® (Newmarket, UK). Drops consisted of 400 nL formed by mixing equal volumes of protein solution and crystallization solution. Crystals were obtained in 2.4 m sodium malonate and 0.1 m BIS‐TRIS propane pH 7.0 after 2 weeks. Crystals were cryo‐protected in the crystallization condition and flash cooled under liquid nitrogen. A complete X‐ray data set was collected at I24 beam line of Diamond Light Source at wavelength of 0.9686 Å and using a Pilatus detector under 100 K. Data were processed, indexed and scaled using XDS 32, POINTLESS 33 and AIMLESS 34 with the autoPROC pipeline 35. Resolution bins with CC1/2 above 0.5 and <*I*>/<σ(*I*)> above 2 were used to obtain maximum acceptable resolution. Initial phases were calculated using the molecular replacement program Phaser. The coordinates of human frataxin (PDB code 3s4m) were used as the search model. Subsequently, the initial model generated by Phaser was refined through an iterative cycle using coot 36 and refmac5 37. The final model structures were validated using the Molprobity server 38 at http://molprobity.biochem.duke.edu. The data processing and refinement statistics are available in Table 2. The coordinates are available in PDB with the accession code 6FCO.

### Solution studies by NMR

All experiments were recorded at 37 °C on Bruker instruments with cryogenically cooled triple resonance probes. The resonances of Ct‐frataxin were identified according to the previously reported assignment 18. A 3D ^15^N NOESY‐HSQC spectrum was acquired at 800 MHz to check for sequential HN–HN connectivities. NMR titration experiments were carried to characterise the structural and functional aspects of Ct‐frataxin protein in apo, ligand and protein bound states. The unlabeled reaction substrates (NH_4_)_2_Fe(SO_4_)_2_, Fe_2_(SO_4_)_3_ and the IscU and IscS proteins were titrated individually into ^15^N labeled samples of Ct‐frataxin. The progress of the titration was monitored by recording one‐dimensional ^1^H and two‐dimensional ^1^H–^15^N HSQC correlated spectra until no further changes in chemical shifts were detected in the ^1^H–^15^N HSQC spectrum of Ct‐frataxin protein. The titrations were recorded at 600, 700 or 950 MHz collecting a total of either 2048 × 128 or 2048 × 256 data points in the ^1^H and ^15^N dimensions and 8–16 scans per *t*_1_ increment complex point. ^15^N relaxation measurements including ^15^N longitudinal (*R*_1_) and transverse (*R*_2_) relaxation rate constants, and {^1^H}^15^N hNOEs were recorded at 800 MHz on a Ct‐frataxin 1 mm sample, using established pulse sequences 39, 40. Peak intensity errors were estimated from selected data sets with replicated relaxation delays. Spectra were acquired at 37 °C, using 2048 × 128 complex points in the *t*_2_ × *t*_1_ dimensions at ^1^H frequencies of 800 MHz. The spectral widths in the direct and the indirect dimensions were 12820.51 × 2717.05 Hz at ^1^H proton frequencies of 800 MHz. All NMR data were processed using NMRpipe package and analyzed in ccpn analysis software 41 (http://www.ccpn.ac.uk/).

### Enzymatic assays

All enzymatic assays were performed in an anaerobic chamber (Belle Technology, Weymouth, UK) under nitrogen atmosphere. The kinetics of cluster formation on IscU were followed at 456 nm as a function of time by absorbance spectroscopy using a Cary 50Bio Varian spectrophotometer (Palo Alto, CA, USA). The initial rates were measured by incubating 1 μm IscS, 50 μm IscU, 250 μm Cys, 3 mm DTT and 25 μm Fe^2+^ in 50 mm Tris‐HCl at pH 8.0 and 150 mm NaCl in the absence or presence of different molar rations (0–50 μm) of Ct‐frataxin. The reactions were initiated after half an hour incubation by addition of 1 μm IscS and 250 μm Cys. Each measurement was repeated at least three times on independent batches of proteins.

## Conflict of interest

The authors declare no conflict of interest.

## Author contributions

MR produced the protein and the NMR analysis, MJ crystallized and solved the X‐ray structure; RP carried out the enzymatic assays; RY helped the spectrum assignment; EC supervised the structure determination; AP analyzed the results, wrote the paper, and supervised the project.
